# Tentacular-Type Esophageal Papilloma

**DOI:** 10.14309/crj.0000000000001521

**Published:** 2024-09-27

**Authors:** Zaid Ansari, Pablo A. Bejarano, Asad Ur Rahman

**Affiliations:** 1 Department of Gastroenterology, Cleveland Clinic Florida, Weston, FL; 2 Department of Pathology, Cleveland Clinic Florida, Weston, FL

## CASE REPORT

**Figure 1.**
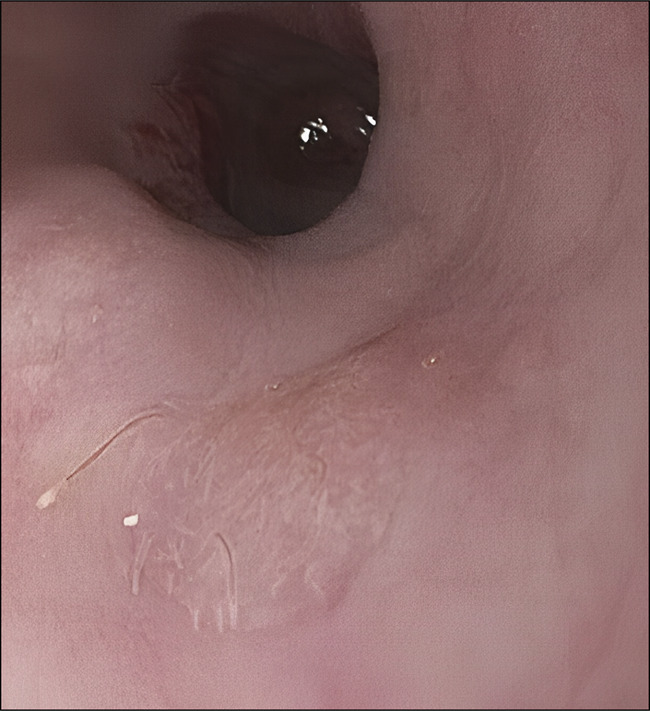
Upper endoscopy under white light showed sessile lesions with filiform protuberances.

**Figure 2.**
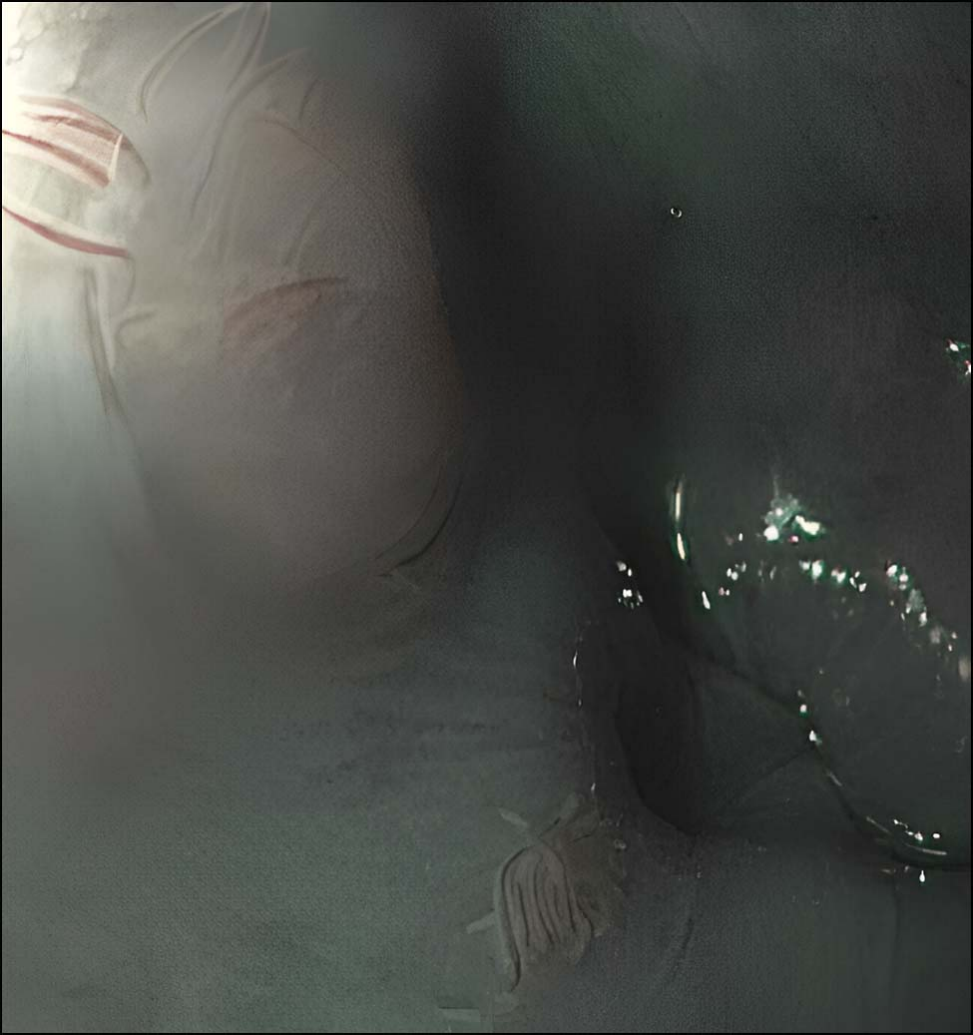
Narrow band imaging highlighting these tentacle-like processes.

**Figure 3.**
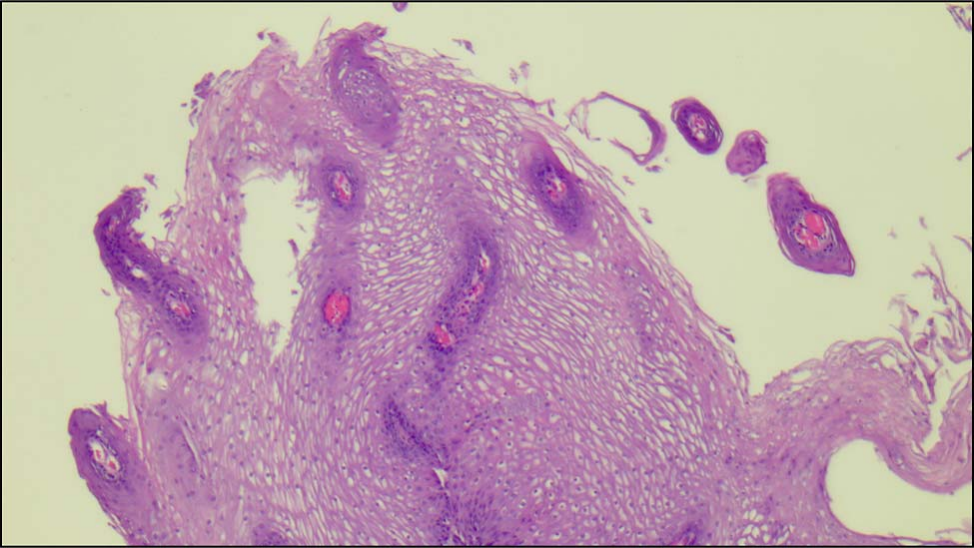
Histopathological specimen (100×).

A 54-year-old woman with a medical history of irritable bowel syndrome underwent an upper endoscopy due to new symptoms of dysphagia to solids greater than liquids. Two plaques, 5–6 mm in size, with some filiform protuberances were found in the middle third of the esophagus, 23–25 cm from the incisors (Figure 1). Narrow band imaging was performed, which did not show any abnormal vasculature (Figure 2). Histopathology showed esophageal mucosa with thin papilloma (Figure 3). Esophageal papilloma is classified as a benign lesion with low potential for malignancy.^1^ Although the etiology is unclear, chronic mucosal irritation and human papillomavirus may be associated. Our patient has an uncommon gross appearance with filiform/tentacle-like protuberances that has only been reported once previously in literature.^2^

## DISCLOSURES

Author contributions: Z. Ansari: drafting the manuscript, revisions, provided and cared for study patient. PA Bejarano and AU Rahman: review of manuscript, final draft, provided and cared for study patient. Z. Ansari is the article guarantor.

Financial disclosure: None to report.

Informed consent was obtained for this case report.

## References

[1] Ahmad AI Lee A Nithagon P Esophageal squamous papilloma: Literature review and case-control retrospective study with histopathological exam of human papillomavirus. JGH Open. 2023;7(10):674–81. 37908288 10.1002/jgh3.12942 PMC10615170

[2] Zimmer V. Tentacular-type esophageal squamous papilloma. Mayo Clin Proc. 2019;94(8):1551. 31378230 10.1016/j.mayocp.2019.03.025

